# Recent advances in understanding and treating nephrotic syndrome

**DOI:** 10.12688/f1000research.10165.1

**Published:** 2017-02-09

**Authors:** Agnieszka Bierzynska, Moin Saleem

**Affiliations:** 1 University of Bristol, Bristol Royal Hospital for Children, Bristol, UK

**Keywords:** idiopathic nephrotic syndrome, minimal change nephrotic syndrome, MCNS, focal segmental glomerulosclerosis, FSGS, SRNS

## Abstract

Idiopathic nephrotic syndrome (INS) is one of the most common glomerular diseases in children and adults, and the central event is podocyte injury. INS is a heterogeneous disease, and treatment is largely empirical and in many cases unsuccessful, and steroids are the initial mainstay of therapy. Close to 70% of children with INS have some response to steroids and are labelled as steroid-‘sensitive’, and the rest as steroid-‘resistant’ (also termed focal segmental glomerulosclerosis), and single-gene mutations underlie a large proportion of the latter group. The burden of morbidity is enormous, both to patients with lifelong chronic disease and to health services, particularly in managing dialysis and transplantation. The target cell of nephrotic syndrome is the glomerular podocyte, and podocyte biology research has exploded over the last 15 years. Major advances in genetic and biological understanding now put clinicians and researchers at the threshold of a major reclassification of the disease and testing of targeted therapies both identified and novel. That potential is based on complete genetic analysis, deep clinical phenotyping, and the introduction of mechanism-derived biomarkers into clinical practice. INS can now be split off into those with a single-gene defect, of which currently at least 53 genes are known to be causative, and the others. Of the others, the majority are likely to be immune-mediated and caused by the presence of a still-unknown circulating factor or factors, and whether there is a third (or more) mechanistic group or groups remains to be discovered. Treatment is therefore now being refined towards separating out the monogenic cases to minimise immunosuppression and further understanding how best to stratify and appropriately direct immunosuppressive treatments within the immune group. Therapies directed specifically towards the target cell, the podocyte, are in their infancy but hold considerable promise for the near future.

## Introduction

The glomerulus is the filtration unit of the kidney and allows the passage of vast amounts of water and small solutes (180 l per day in an adult human) into the urinary space while preventing the passage of almost any protein (<30 mg/day). This highly specialised property is achieved by the glomerular filtration barrier (GFB), comprising endothelial cells, a unique basement membrane, and podocytes on the urinary side.

Damage to the GFB can come from many potential sources, including genetic defects (primarily affecting podocytes), paracrine events (for example, affecting endothelial-podocyte cross-talk or from adjacent mesangial cells), or systemic circulating insults. The last of these can take many forms, including circulating immune complexes, metabolic disturbances (most commonly, diabetes), infections, toxins (for example, shiga toxin in haemolytic uraemic syndrome) and drugs.

The clinical manifestation is of oedema, low serum albumin, and massive proteinuria. This leads to numerous consequences, related to circulatory/dynamic effects, and loss of essential circulating proteins.

Idiopathic, or primary, nephrotic syndrome is often used to describe the group of patients for whom no specific cause has been identified, and the histology is relatively non-specific. These patients will usually receive immunosuppression without knowledge of the mechanism and be categorised according to response. So the challenge is to understand and categorise the underlying injury at a molecular level and therefore adapt treatments according to the likely mechanism.

## Idiopathic nephrotic syndrome current classification

Current classification of idiopathic nephrotic syndrome (INS) is based on observational characteristics. This can be according to response to steroids, which is the usual first-line response, or according to light microscopy patterns of injury on renal biopsy. The commonest biopsy finding, particularly in children, is of ‘minimal change’ nephrotic syndrome (MCNS). This means that light microscopy is entirely normal, importantly not even showing any signs of immune infiltration or upregulation of immune markers. Pathology is seen only at the level of electron microscopy, where effacement or flattening of podocyte foot processes is the characteristic finding in any patient with nephrosis. The next most common biopsy finding (between 10% and 20% of patients) is of focal segmental glomerulosclerosis (FSGS), a description of chronic, fibrotic damage in the glomerulus. Response to therapy often (but not always) correlates with these biopsy findings, in that most patients with MCNS will be sensitive to steroids and most with FSGS will be resistant. However, there are many shades in between these phenotypes, and patients frequently become relapsing, steroid-dependent, calcineurin-responsive, and so on. This reflects our lack of knowledge of the underlying mechanisms and the need for a different approach to this disease.

## Genetics

The most concrete advance in establishing the underlying mechanistic cause of INS in recent years has been the discovery that a substantial proportion of patients with steroid-resistant nephrotic syndrome (SRNS) have a single-gene mutation causing their disease ( Table 1). To date, at least 53 different genes have been implicated ^1^, almost all causing structural or functional defects in the podocyte. Some mutations cause isolated kidney disease, others are part of a syndromic condition. Most are autosomal-recessive, though a few present as X-linked or autosomal-dominant, the latter usually presenting later in life. Some genes have been linked with steroid or cyclosporine sensitivity, such as *EMP2* ^2^ or *KANK* genes ^3^, though the data are still sporadic and need consistent verification in other pedigrees.

**Table 1.** Some of the more common genes mutated in monogenic steroid-‘resistant’ nephrotic syndrome.

Function	Protein	Gene	Syndrome	Mode of inheritance	Histology
Slit diaphragm protein	Nephrin	*NPHS1*	Congenital nephrotic syndrome	AR	Microcystic dilatation of tubules and progressive mesangial sclerosis
			SRNS	AR	MPGN, MCD, FSGS
	Podocin	*NPHS2*	Congenital nephrotic syndrome	AR	
			SRNS	AR	FSGS
	PLCE1	*NPHS3*	DMS	AR	DMS
			SRNS	AR	FSGS
	CD2AP	*CD2AP*	SRNS	AD/AR	FSGS
SD ion channel	TRPC6	*TRPC6*	SRNS	AD	FSGS
Developmental	WT1	*WT1*	Denys–Drash syndrome	AD	DMS
			Frasier syndrome	AD	FSGS
			Isolated SRNS	AR	DMS, FSGS
Actin regulating	α-Actinin 4	*ACTN4*	Adult-onset SRNS	AD	FSGS
	Inverted formin 2	*INF2*	SRNS	AD	FSGS
Glomerular basement membrane	Laminin β2	*LAMB2*	Pierson syndrome	AR	DMS, FSGS
Others	tRNA-LEU	*MTTL1*	MELAS	Maternal	FSGS
	Parahydroxybenzoate- polyprenyltransferase	*COQ2*	CoQ10 deficiency	AR	Collapsing glomerulopathy
	Prenyl diphosphate synthase subunit 2	*PDSS2*	CoQ10 deficiency/ Leigh syndrome	AR	FSGS
	SMARCA-like protein	*SMARCL1*	Schimke immuno- osseous dysplasia	AR	FSGS
	Lysosomal integral membrane protein type 2	*SCARB2*	Action myoclonus- renal failure syndrome	AR	
	LIM homeobox transcription factor 1β	*LMX1B*	Nail patella syndrome	AD	FSGS
	Zinc metallo-proteinase STE24	*ZMPSTE24*	Mandibuloacral dysplasia	AR	
	WD repeat-containing protein 73	*WDR73*	Galloway–Mowat syndrome	AR	DMS, FSGS
	Phosphomannomutase 2	*PMM2*	Congenital defects of glycosylation	AR	Collapsing glomerulopathy
	β-1, 4-Mannosyltransferase	*ALG1*	Congenital defects of glycosylation	AR	FSGS

AD, autosomal-dominant; AR, autosomal-recessive; CoQ10, coenzyme Q _10_; DMS, diffuse mesangial sclerosis; FSGS, focal segmental glomerulosclerosis; MCD, minimal change disease; MELAS, mitochondrial myopathy, encephalopathy, lactic acidosis and stroke-like episodes; MPGN, membranoproliferative glomerulonephritis; SRNS, steroid-‘resistant’ nephrotic syndrome.

The incidence of genetic disease in the population varies with age, and over 80% of patients presenting under the age of a year have an identifiable mutation ^4, 5^. Overall, in childhood, the incidence in an unselected UK national cohort of SRNS has been reported as 26.5% ^1^. The incidence in adulthood remains to be ascertained, and phenotype even within individual pedigrees, such as *ACTN4* mutations ^6^, is often highly variable.

With the advent of next-generation sequencing, the ability to screen for genetic causes in a clinical setting, despite the ever-increasing number of genes to be screened, has become practical, cost-effective and rapid ^7^ and is changing clinical practice. Gene panels such as the Bristol SRNS panel ( https://www.nbt.nhs.uk/severn-pathology/pathology-services/bristol-genetics-laboratory-bgl) can now yield a result within 4 weeks, thus potentially obviating the need for a biopsy and allowing consideration of early withdrawal of immunosuppression if a positive result is found.

## Circulating factor disease

Patients who screen negative for the known SRNS genes, as well as those with steroid-sensitive disease, fall into a category wherein a substantial proportion will have an immune-mediated, circulating factor disease (CFD). The evidence for this remains circumstantial, and the most compelling clinical scenario is that of post-transplant recurrence of disease ^8^. This is a situation where patients with SRNS who eventually reach established renal failure are then transplanted. Between 30% and 50% of patients suffer from rapid (most commonly, hours or days post-transplant) recurrence of massive proteinuria, and biopsy of the new kidney shows classic foot process effacement. This is presumed to be due to a circulating factor, and a fascinating recent case report described a patient with early recurrence, whose newly transplanted kidney was then removed and re-transplanted into a recipient without SRNS, and the kidney recovered completely ^9^. It is not known where the circulating factor or factors originate, but the fact that immunosuppression is often effective, and relapses are often triggered by viral infections, points to the immune system ( Table 2). Both T cells and B cells have been implicated ^10^; for example, anti-CD20 B cell-depleting monoclonal antibodies are highly effective in some patients ^11^, and a T helper 17/regulatory T (Th17/Treg) cell imbalance has been described in minimal change disease ^12^. Another clue to a circulating factor pathogenesis in steroid-sensitive nephrotic syndrome, where the biopsy shows minimal change, is the lack of immune cell infiltration or activation within the glomerulus itself.

**Table 2.** A selection of suggested circulating factors in idiopathic nephrotic syndrome.

Postulated circulating factor	Experimental or clinical evidence
Hemopexin	Both recombinant and human hemopexin induced reversible proteinuria in rats ^32^. Decreased serum hemopexin with increased protease activity in ‘minimal change’ nephrotic syndrome (MCNS) in relapse ^33^. Induced nephrin-dependent cytoskeletal rearrangements in podocytes ^34^
suPAR (soluble urokinase-type plasminogen activator receptor)	Activated podocyte β3 integrin, resulting in reorganisation of the actin cytoskeleton High levels were reported in patients with focal segmental glomerulosclerosis (FSGS) and post-transplant recurrence ^35^. Clinical data were not consistently replicated in other studies ^36^.
Tumour necrosis factor-alpha (TNFα)	Increased concentrations were found in culture supernatants of mitogen-stimulated peripheral blood mononuclear cells from patients with FSGS ^37^. Four of ten children with primary FSGS exhibited remission of proteinuria in a phase I trial of a TNFα-neutralising antibody ^38^.
Interleukin-13 (IL-13)	Increased expression of mRNA and cytoplasmic IL-13 in CD4 ^+^/CD8 ^+^ T cells from children with steroid-sensitive idiopathic nephrotic syndrome ^39^. Overexpression of IL-13 in rats induces MCNS-like disease ^40^.
Galactose (inhibitor of circulating factor)	Binds to putative circulating factor and inhibits its activity ^41^. A clinical trial of galactose showed no remission effect ^42^.

A significant gap in our knowledge is whether there is one common circulating factor that causes all forms of the disease, or several distinct factors, or possibly a family of related factors. There are no indications at present, either laboratory or clinical, that help to resolve this question, though we have made progress in some areas of prediction of CFD.

## Clinical biomarkers

There have been no consistent clinical cues to either whether a patient with nephrotic syndrome has the risk of becoming steroid-resistant in the future or whether they will suffer recurrence post-transplant. There are weak clinical associations with recurrence (for example, age at onset of disease ^13, 14^, race ^15^, serum albumin at diagnosis ^13^ or time to first dialysis/transplant ^13, 15, 16^). Interestingly, the last two features may point to the possibility that CFD has a more aggressive presentation and natural course, compared with the monogenic or ‘other’ groups. To address the question of whether there are clinical features that pertain to CFD, we hypothesised that patients with the archetypal CFD, those with post-transplant recurrence, would have distinct early clinical features regarding their initial response to immunosuppression. If a patient has initial steroid sensitivity (otherwise described as secondary steroid resistance), they are likely to have an immune-mediated circulating factor causing their underlying disease and therefore high risk of recurrence. On retrospective review of 150 transplanted patients with SRNS, we found that 93% of patients with initial steroid sensitivity recurred post-transplant (odds ratio = 30, *P* <0.0001), making this by far the strongest clinical biomarker for recurrent disease yet found ^17^.

## Laboratory biomarkers

Over many decades, researchers have searched for the elusive circulating factor or factors, but there has been no consistent answer to date ^18^. Clearly, if the factor were identified, this would be the ideal biomarker to both diagnose and monitor disease activity. There are other rational ways to approach biomarker discovery, based on the fact that circulating blood from patients with active disease is likely to carry the active factor and that we know the most likely target cell of such a factor, the podocyte. Therefore, an approach of stimulating human podocytes *in vitro* with plasma or serum from disease has been used by ourselves and others, and podocyte damage has been assayed in various ways. For example, we have shown that plasma exchange fluid from patients being treated for post-transplant recurrence causes relocalisation of key podocyte slit diaphragm proteins ^19^ and abnormal signalling and cell motility ^20^. Torban *et al*. showed a tumour necrosis factor-alpha (TNFα) pathway-dependent change in podocyte cytoskeletal changes ^21^ and also changes in podocyte focal adhesion complexes ^22^. The challenge is to show consistency and disease specificity by using these types of assays, before they can be introduced into clinical practice.

## Newer therapies

The mainstay of current therapy is immunosuppression, which is appropriate for the immune-mediated group of diseases, but there is very limited evidence of efficacy in monogenic disease. There are studies that support a direct effect of some immunosuppressive drugs on the podocyte ^23– 27^, though the majority of clinical evidence points to efficacy being achieved via effects on the immune system.

Some newer therapies have been proposed on the basis of direct targeting of either the immune system or podocyte signalling pathways. The most prominent of these are the use of anti-CD20 monoclonal antibodies ^28^, which deplete B cells, and anti-B7-1 monoclonal antibody therapy ^29^. The latter has been proposed following the observation that in certain experimental and human glomerular diseases the T-cell co-stimulatory molecule B7-1 has been noted to be upregulated on podocytes ^30^. This can be targeted by the drug abatacept and is the subject of current trials in larger numbers of patients.

## Future stratification and personalised medicine

Our understanding of INS at the molecular, cell biology and genetic levels is advancing rapidly, and the information gained will be critical in stratification and re-categorisation of patients into clinically useful mechanistic categories. The advent of rapid second- and now third-generation sequencing technologies is already changing clinical practice. Exome/genome sequencing, side by side with powerful population sequencing databases such as the exome aggregation consortium (ExAC) ^31^, means that novel genes will continue to be discovered, even from sporadic cases, to complete our knowledge of the extent of heritable disease in this population.

A challenge of CFD is to discover whether this is a single entity, or separate mechanistic diseases, and target the most appropriate therapies to the individual groups. For example, is relapsing non-progressive disease different from secondary steroid resistance, which usually leads to renal failure (and recurrence post-transplant)?

Novel therapies based on common podocyte signalling pathways are already on the horizon, and the availability of large national patient registries currently being developed will greatly accelerate the ability to trial these in appropriately chosen patient groups.
